# Supplementary material for: Transient ice ring observed during the 15 January 2022 eruption of Hunga volcano
Source: Commun Earth Environ. 2025 Nov 14;6(1):901. doi: 10.1038/s43247-025-02875-0 (PMC12618230; doi:10.1038/s43247-025-02875-0)
Supplement: Supplementary file 1 — Supplementary material [file 43247_2025_2875_MOESM1_ESM.pdf]

Supplementary material for "Transient ice ring observed during the 15 January 2022 eruption of Hunga volcano"

Andrew T. Prata[1,2*], Roy G. Grainger[3], Isabelle A. Taylor[4] and Alyn Lambert[5]

[1]Sub-Department of Atmospheric, Oceanic and Planetary Physics, Clarendon Laboratory, Parks Road, Oxford, OX1 3PU, UK.
[2]Now at: CSIRO Environment, Research Way, Clayton, Victoria 3168, Australia.
[3]National Centre for Earth Observation, Atmospheric, Oceanic and Planetary Physics, University of Oxford, Parks Road, Oxford, OX1 3PU, UK.
[4]COMET, Atmospheric, Oceanic and Planetary Physics, University of Oxford, Parks Road, Oxford, OX1 3PU, UK.
[5]Jet Propulsion Laboratory, California Institute of Technology, Pasadena, California, USA.

*Corresponding author(s). E-mail(s): andrew.prata@csiro.au; Contributing authors: r.grainger@physics.ox.ac.uk; isabelle.taylor@physics.ox.ac.uk; alyn.lambert@jpl.nasa.gov;

Supplementary Movie 1

This movie shows the 8.6 - 11 μm brightness temperature difference for all available 10-minute Himawari-8 data from 15:17 UTC on 13 January 2022 to 06:07 UTC on 15 January 2022. Hunga volcano is indicated as a red triangle. Note that times annotated on the movie are nominal Himawari times but the actual observation time at the location of Hunga has an offset of ∼7 minutes from the nominal scan time (e.g. 15:00 UTC is actually closer to 15:07 UTC).

Supplementary Movie 2

This movie shows an animation of the data presented in Fig. 1 of the main text from 04:07 UTC to 07:07 UTC on 15 January 2022 (see Fig. 1 caption for details).

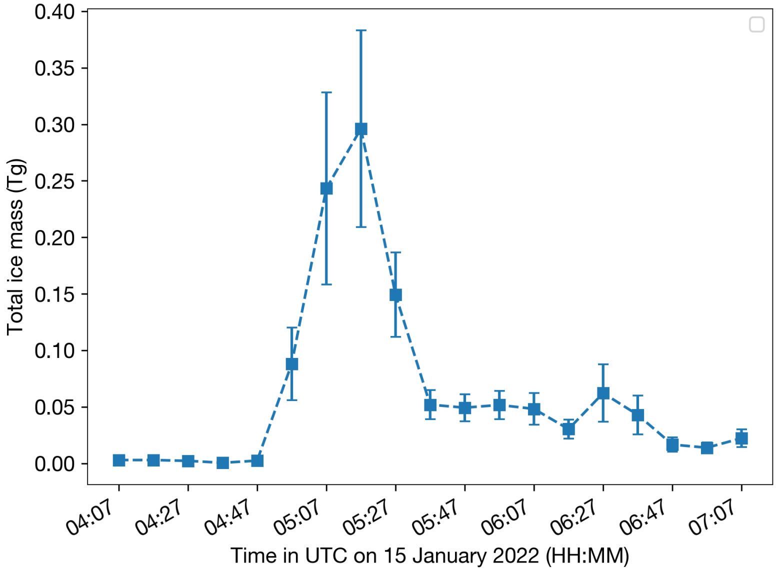

Supplementary Figure 1 Total ice mass time series calculated from the ORAC satellite retrievals. Note that the total ice mass timeseries shown here includes any retrieval with an 8.6 - 11 μm brightness temperature difference greater than or equal to 15 K and is different to the 'ice-ring region' referred to in the main text (i.e. the annulus between 250–300 km centred on the volcano).

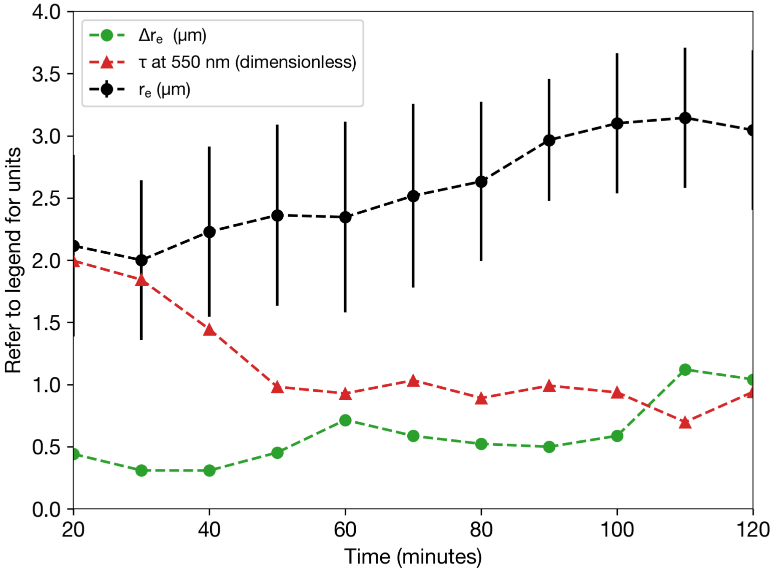

Supplementary Figure 2 Time series of ORAC satellite retrievals in the ice ring region (refer to main text for how this is defined). Here r_e and error bars refer to the mean effective radius and standard deviation, respectively (as in Fig. 5 of the main manuscript), Δr_e is the mean absolute uncertainty derived from the optimal estimation algorithm which provides uncertainties on a per-pixel basis and τ is the mean optical depth at 550 nm. The time is calculated in minutes relative to 04:47 UTC on 15 January 2022.
