# Supplementary material for: Transient ice ring observed during the 15 January 2022 eruption of Hunga volcano
Source: Commun Earth Environ. 2025 Nov 14;6(1):901. doi: 10.1038/s43247-025-02875-0 (PMC12618230; doi:10.1038/s43247-025-02875-0)
Supplement: Supplementary file 2 — Description of Additional supplementary file [file 43247_2025_2875_MOESM2_ESM.pdf]

Description of Additional Supplementary Files

File name- Supplementary Movie 1

Description-

This movie shows the 8.6 - 11 μm brightness temperature difference for all available 10-minute Himawari-8 data from 15:17 UTC on 13 January 2022 to 06:07 UTC on 15 January 2022. Hunga volcano is indicated as a red triangle. Note that times annotated on the movie are nominal Himawari times but the actual observation time at the location of Hunga has an offset of ~7 minutes from the nominal scan time (e.g. 15:00 UTC is actually closer to 15:07 UTC).

File name- Supplementary Movie 2

Description-

This movie shows an animation of the data presented in Fig. 1 of the main text from 04:07 UTC to 07:07 UTC on 15 January 2022 (see Fig. 1 caption for details).
